# Ethics in Patient Preferences for Artificial Intelligence–Drafted Responses to Electronic Messages

**DOI:** 10.1001/jamanetworkopen.2025.0449

**Published:** 2025-03-11

**Authors:** Joanna S. Cavalier, Benjamin A. Goldstein, Vardit Ravitsky, Jean-Christophe Bélisle-Pipon, Armando Bedoya, Jennifer Maddocks, Sam Klotman, Matthew Roman, Jessica Sperling, Chun Xu, Eric G. Poon, Anand Chowdhury

**Affiliations:** 1 Department of Medicine, Duke University School of Medicine, Durham, North Carolina; 2 Department of Biostatistics & Bioinformatics, Duke University School of Medicine, Durham, North Carolina; 3 Center for Bioethics, Harvard Medical School, Boston, Massachusetts; 4 Faculty of Health Sciences, Simon Fraser University, Burnaby, British Columbia, Canada; 5 Department of Health Technology Solutions, Duke University Health System, Durham, North Carolina; 6 Duke Clinical & Translational Science Institute, Duke University, Durham, North Carolina

## Abstract

**Importance:**

The rise of patient messages sent to clinicians via a patient portal has directly led to physician burnout and dissatisfaction, prompting uptake of artificial intelligence (AI) to alleviate this burden. It is important to understand patient preferences around AI in patient-clinician communication as ethical guidelines on appropriate use and disclosure (patient notification of AI use) are developed.

**Objective:**

To analyze patient preferences regarding use of AI in electronic messages.

**Design, Setting, and Participants:**

A survey study was conducted within the Duke University Health System’s patient advisory committee, consisting of individuals 18 years or older who participate in periodic surveys to inform health system patient care practices. Multiple surveys were administered to test the impact of different factors, including response author, disclosure (AI, human, or none), and seriousness of the topic. A follow-up survey assessed preferred disclosure verbiage. Surveys were administered from October 31 to December 11, 2023.

**Exposure:**

Multiple surveys.

**Main Outcomes and Measures:**

Participants rated their overall satisfaction, usefulness of the information, and perceived level of care on a 5-point Likert scale.

**Results:**

Of the 2511 members surveyed, 1455 (57.9%) responded, with respondents being older (median age, 57 [IQR, 49-70] vs 53 [IQR, 41-62] years), more educated (872 of 1083 [80.5%] vs 319 of 440 [72.5%] with a college or graduate degree), and predominantly female (921 [63.3%]). Participants preferred AI- compared with human-drafted responses, with a mean difference for satisfaction of −0.30 (95% CI, −0.37 to −0.23) points, usefulness of −0.28 (95% CI, −0.34 to −0.22) points, and perception they were cared for of −0.43 (95% CI, −0.50 to −0.37) points. Participants tended to have higher satisfaction with a human disclosure over AI disclosure, with a mean difference of 0.13 (95% CI, 0.05-0.22) points, and with no disclosure over AI authorship disclosure, with a mean difference of 0.09 (95% CI, 0.01-0.17) points. Regardless of author or disclosure type, more than 75% of respondents were satisfied (agree or strongly agree) with the response.

**Conclusions and Relevance:**

In this survey study, participants expressed a mild preference for messages written by AI but had a slightly decreased satisfaction when told AI was involved. Patient experience must be considered along with ethical implementation of AI. Although AI disclosure may slightly reduce satisfaction, disclosure should be maintained to uphold patient autonomy and empowerment.

## Key Points

**Question:**

How do patients feel about the use of artificial intelligence (AI) to draft responses in patient-clinician portal messages?

**Findings:**

This survey study of 1455 respondents showed that while overall satisfaction was high (>75%) regardless of author, respondents preferred responses written by AI over those written by a human (mean difference, 0.30 points on a 5-point Likert scale for satisfaction). However, when an AI author was disclosed, satisfaction was lower for AI compared with a human author (mean difference, 0.13 points).

**Meaning:**

Reduced satisfaction due to AI disclosure should be balanced with the importance of patient autonomy and empowerment.

## Teaser

This survey study analyzes patient preferences around the use of artificial intelligence vs humans in generating responses to patient portal messages.

## Introduction

The rise of electronic communication sent to clinicians via the patient portal has directly led to clinician burnout and dissatisfaction.^1,2^ With patients increasingly messaging their clinicians, replying to in-basket messages (akin to email) has become a burdensome task consisting of medical questions, refill requests, and administrative and scheduling requests.^3^ As large-language models (LLMs) and artificial intelligence (AI) become widely available, health care leaders have sought automated solutions that reduce the time burden on physicians and advanced practice clinicians while maintaining high-quality, empathetic care for patients.

Software companies, including electronic health record (EHR) companies, are partnering to integrate generative AI technology into the EHR to address the in-basket problem.^4^ Health systems have already started implementing AI in-basket solutions, with one system showing measurable reduction in task load burden and burnout as measured by clinician surveys.^5^ Liu et al^6^ demonstrated an LLM’s ability to generate responsive, empathetic, and accurate responses to patient questions as evaluated by clinicians. Similarly, a small team of health care professionals preferred AI-drafted responses to medical questions over physician responses, but preferences of patients were not assessed.^7^ Research on patient preferences regarding AI tools is thus far lacking in the literature.

Without understanding patient preferences around AI in patient-clinician messages, appropriate use, governance, and disclosure policies are difficult to develop and, at best, incomplete. Industry-agnostic AI oversight, which in many cases currently recommends disclosing AI to impacted audiences, may need to be modified or tailored for communication directly with patients, a particularly sensitive and potentially high-risk use case.^8,9^ From a bioethics view, Blumenthal-Barby^10^ wrote, “While I agree in principle with the benefits of transparency and disclosure, in the context of health care AI and machine learning, it is unclear how and why notice and expatiation of use of an AI-based system ought to be achieved with patients.” Without research around the impact of disclosure on patient satisfaction, we are left with ethical and operational questions for health systems using AI as a tool to support the in-basket.

To ethically use AI to address the in-basket problem, it is important to understand how patients feel about the use and disclosure of AI tools in communication. How does disclosure impact patient satisfaction? If impacted, how should patient satisfaction be weighed against the ethical dilemmas raised by inherent power imbalance between patients and clinicians? How, if at all, should we disclose use of AI?

We aimed to analyze patient preferences around the use of AI in generating responses to patient portal messages to better inform this discussion. We hypothesized that patient preferences would vary based on AI vs human disclosure and the response author and would be influenced by the seriousness of the patient’s message.

## Methods

### Ethics

We conducted a series of surveys among patients from our voluntary, uncompensated patient advisory committee in which individuals 18 years or older participate in periodic surveys to inform patient care practices at Duke University Health System (DUHS). Participants sign an informed consent to join the patient advisory committee. This survey study was determined to be exempt by the Duke Health Institutional Review Board and has been preregistered with ClinicalTrials.gov.^11^ We adhered to the American Association for Public Opinion Research (AAPOR) reporting standards for survey studies.

### Participant Population

This study was conducted at DUHS, a medium-sized health system in Durham, North Carolina, between October 31 and December 11, 2023. The advisory committee consists primarily of past and current DUHS patients (96%), plus a small minority of community members who elect to serve (4%). Members provide input on various topics related to health care, patient experience, and service enhancements. Demographic information is collected from all participants at the time they join the committee. All participants were sent the initial series of surveys with participation being fully voluntary. The follow-up survey on preferred disclosure statements was sent only to those who responded to the initial survey.

### Survey Design

We first conducted a series of randomized full-factorial (18 total combinations) surveys to test 3 different factors that could potentially impact outcomes related to patient satisfaction, with each participant receiving 3 scenarios. We then sent a follow-up survey about preferred disclosure verbiage.

### Experimental Factors

The 18 survey scenarios (eTables 1 and 5 and eFigure 1 in Supplement 1) differed based on 3 factors: (1) the content of the 3 hypothetical patient’s messages, which varied by the seriousness of the clinical topic; (2) the response text, which differed by the author (AI or human); and (3) the disclosure, which was categorized as AI, human, or none. Both concordant and discordant combinations were included (eg, a human response was paired with a human disclosure, an AI disclosure, and no disclosure). The 3 clinical topics were a routine medication refill request (low seriousness), a question about an adverse effect of medication (moderate seriousness), and a potential malignant neoplasm on imaging findings (high seriousness). The degree of seriousness was agreed on by the study team. The human responses and both disclosures were written and reviewed by a multidisciplinary team of 3 physicians with outpatient experience who were given each patient question and asked to write a realistic response based on how they typically draft responses to patients. The generative AI responses were written using an LLM (GPT 3.5; OpenAI) and were reviewed for accuracy by this team of study physicians (J.S.C. and A.C.), who made minimal changes to the responses. The AI responses concerning topics of low and moderate seriousness were written using the August 3, 2023, version of the LLM (ChatGPT; OpenAI), and the topic of high seriousness was written using the September 24, 2023, version.

Participants received a series of 3 surveys over 3 weeks, which contained each topic once in a random order. Participants were randomized, with each receiving a unique combination of topic order, author(s), and disclosure type(s).

### Outcomes

For each electronic survey, participants were asked to read a vignette in which 1 clinical topic, 1 response, and 1 disclosure or lack thereof was provided before the survey questions. Following their review of the vignettes, they were prompted to use a 5-point Likert scale to rate their overall satisfaction, usefulness of the information, and the perception of care (hereinafter expressed as how cared for) they felt during the interaction.^12,13^

### Follow-Up Survey: AI Disclosure Statements

All respondents who responded to at least 1 of the initial 3 experimental surveys received a follow-up survey asking them to rank 5 AI disclosures from most to least preferred. The disclosure versions were written and reviewed by a multidisciplinary team of clinicians and patient experience experts who drafted language intended to disclose AI involvement while making the patient feel comfortable with the use of automated tools.

### Additional Participant Data

Demographic data were abstracted from the advisory committee database. These data included age, gender, race and ethnicity, educational level, and employment status, all of which were self-reported. Race and ethnicity were assessed as potential factors that could modify the perception of AI communication, and our respondents self-identified as Hispanic, non-Hispanic Black, non-Hispanic White, and other, which included free-text answers of Anatolian or Turkish, African American and White, Black and Native American, Cape Verdean, Greek, human race, or adopted. Adopted included being a member of a visible minority group raised Afro-Latino, Indian, Jewish, Latino, Lebanese, Middle Eastern, North African Romani and Palestinian North African, Spanish or Italian or Middle Eastern, Syrian, White, White and Asian, White and Mexican American, or mixed ethnicity and races.

### Statistical Analysis

We described participant demographics based on those who did and did not respond, using means and proportions for continuous and categorical variables, respectively. We first performed descriptive statistics on the responses to the follow-up survey. Next, we regressed each of the 3 responses onto the 3 experimental factors of scenario, author, and disclosure. Given the Likert response, this is interpreted as the mean change in response for different categories. We used a linear mixed model to account for repeated responses by individuals and reported the mean coefficient and 95% CIs.

To test whether the author type and disclosure type interacted to influence preferences, including discordant combinations, we assessed the pairwise interaction between author and disclosure. To test whether the seriousness of the topic discussed in the in-basket message exchange influenced preferences around messages based on disclosure, we assessed the pairwise interaction between seriousness of the topic and disclosure. Finally, we conducted an exploratory analysis assessing potential interactions between each of the 5 demographic factors (gender, race and ethnicity, age, educational level, and employment status) and each of the experimental conditions. To account for the multiple categories, we conducted a likelihood ratio test to assess the significance of the interaction term. Given the large number of tests (n = 45), we performed a Benjamini and Hochberg multiple testing correction and report corrected *P* values, with 2-sided *P* < .05 indicating statistical significance.^14^ All analyses were conducted in R, version 4.1.3 (R Project for Statistical Computing).

## Results

### Demographics

Of the 2511 members surveyed, 1455 (57.9%) responded and 1056 (42.1%) did not. The respondent group was older (median age, 57 [IQR, 49-70] vs 53 [IQR, 41-62] years), more educated (872 of 1083 [80.5%] vs 319 of 440 [72.5%] with a college or graduate degree), and predominantly female (921 [63.3%] female, 517 [35.5%] male, and 17 [1.2%] other gender [including gender fluid/queer, nonbinary, transgender female, transgender male, or other]). Further, the proportion of non-Hispanic White members was higher in the respondent group (914 [62.8%]) compared with nonrespondents (551 [52.2%]). Of all remaining respondents, 67 (4.6%) were Hispanic, 369 (25.4%) were non-Hispanic Black, 48 (3.3%) preferred not to answer, and 57 (3.9%) were other race or ethnicity (Table).

**Table.** Demographics of Participants Surveyed and Respondents

Variable	Participant group, No. (%)		
	Overall (N = 2511)	Nonrespondents (n = 1056)	Respondents (n = 1455)
Age, median (IQR), y	55 (46-67)	53 (41-62)	57 (49-70)
Gender			
Male	855 (34.1)	338 (32.0)	517 (35.5)
Female	1613 (64.2)	692 (65.5)	921 (63.3)
Other^a^	43 (1.7)	26 (2.5)	17 (1.2)
Race and ethnicity			
Hispanic	136 (5.4)	69 (6.5)	67 (4.6)
Non-Hispanic Black	698 (27.8)	329 (31.2)	369 (25.4)
Non-Hispanic White	1465 (58.3)	551 (52.2)	914 (62.8)
Prefer not to answer	93 (3.7)	45 (4.3)	48 (3.3)
Other^b^	119 (4.7)	62 (5.9)	57 (3.9)
Highest level of education^c^			
Graduate degree	619 (40.6)	160 (36.4)	459 (42.4)
College graduate	572 (37.6)	159 (36.1)	413 (38.1)
Some college but no degree	251 (16.5)	85 (19.3)	166 (15.3)
High school graduate	76 (5.0)	34 (7.7)	42 (3.9)
Less than high school	5 (0.3)	2 (0.5)	3 (0.3)
Employment status			
Employed	1442 (57.4)	666 (63.1)	776 (53.3)
Not employed	206 (8.2)	102 (9.7)	104 (7.1)
Retired	620 (24.7)	171 (16.2)	449 (30.9)
Disabled	243 (9.7)	117 (11.1)	126 (8.7)

^a^ Includes gender fluid/queer, nonbinary, transgender female, transgender male, and other.

^b^ Free-text answers included Anatolian or Turkish, African American and White, Black and Native American, Cape Verdean, Greek, human race, or adopted. Adopted included being a member of a visible minority group raised Afro-Latino, Indian, Jewish, Latino, Lebanese, Middle Eastern, North African Romani and Palestinian North African, Spanish or Italian or Middle Eastern, Syrian, White, White and Asian, White and Mexican American, or multiethnic and multiracial.

^c^ Data were missing for 988 participants (440 among nonrespondents and 1083 among respondents).

### Response Author

**Figure 1.**
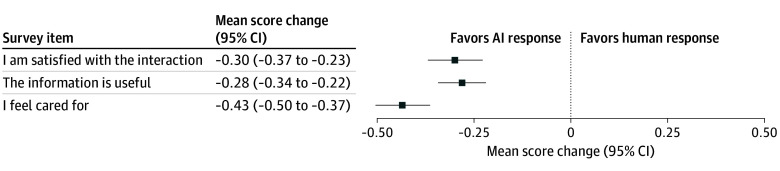
Change in Preference Scores Stratified by Response Author The mean change in a 5-point Likert scale for the responses written by artificial intelligence (AI) vs a human is shown in a forest plot. Tabulated results with *P* values are included in eTable 4 in Supplement 1.

**Figure 2.**
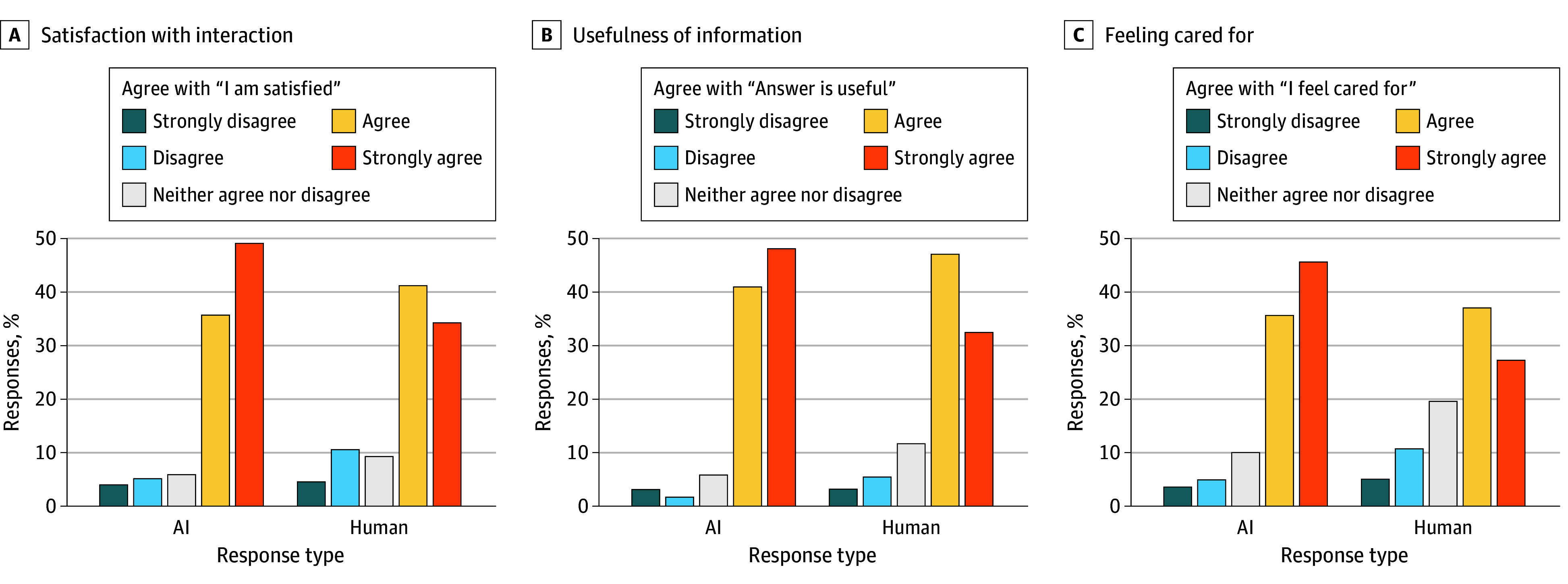
Participant Responses Stratified by Human or Artificial Intelligence (AI) Author

Participants preferred AI-drafted compared with human-drafted responses, regardless of the disclosure or seriousness of the topic. The mean change in the 5-point Likert scale for satisfaction was −0.30 (95% CI, −0.37 to −0.23) points; for usefulness, −0.28 (95% CI, −0.34 to −0.22) points; and for feeling cared for, −0.43 (95% CI, −0.50 to −0.37) points, all favoring AI responses (Figure 1 and eTable 2 in Supplement 1). Most respondents agreed or strongly agreed with the statement “I am satisfied with the interaction,” regardless of response author (ie, 1408 of 1657 [85.0%] for AI and 1241 of 1642 [75.6%] for human) (Figure 2A).

### Disclosure

**Figure 3.**
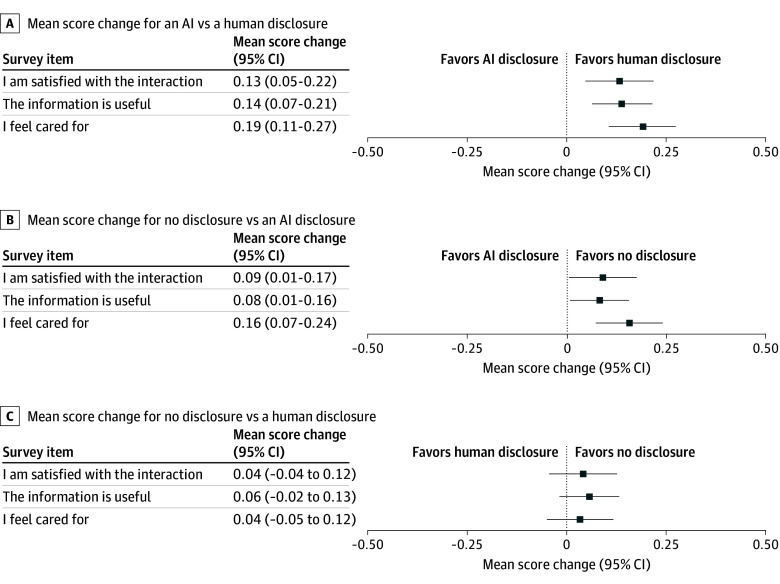
Change in Preference Scores Stratified by Disclosure The mean change in a 5-point Likert scale when the author was disclosed as artificial intelligence (AI), human, or no disclosure is shown in forest plots. Analyses are independent of the actual response author or topic. Tabulated results with *P* values are included in eTable 4 in Supplement 1.

**Figure 4.**
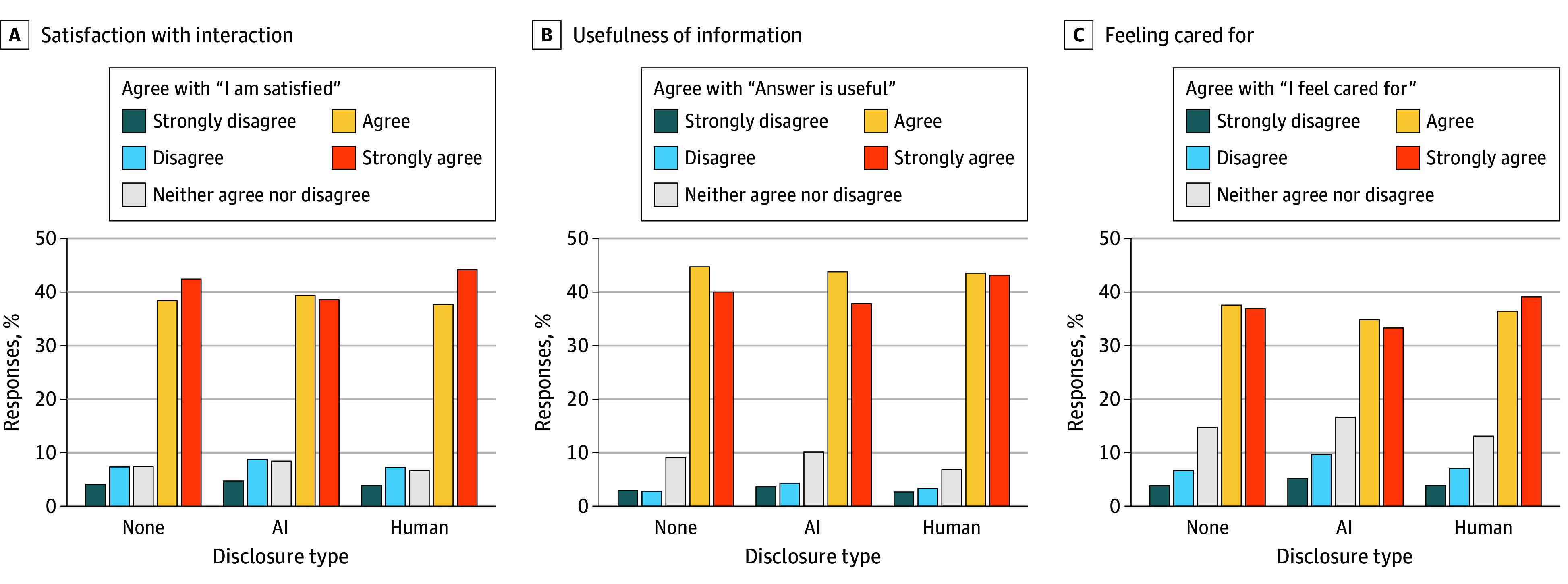
Participant Responses Stratified by Human, Artificial Intelligence (AI), or No Disclosure

Participants preferred messages with a human disclosure or no disclosure compared with an AI disclosure. The mean change in score between AI and human disclosure was 0.13 (95% CI, 0.05-0.22) points for satisfaction, favoring human disclosure, and 0.09 (95% CI, 0.01-0.17) points for satisfaction between AI and no disclosure, favoring no disclosure. Conversely, there was no significant change in preferences when comparing a human disclosure with no disclosure (Figure 3 and eTable 2 in Supplement 1). Most respondents agreed or strongly agreed with the statement “I am satisfied with the interaction,” regardless of disclosure (897 of 1108 [81.0%] for no disclosure, 857 of 1099 [78.0%] for computer disclosure, and 895 of 1092 [82.0%] for human disclosure) (Figure 4).

### Interaction Effects

While participants preferred messages written by AI but human disclosure (or none), there was no observed interaction between response author and disclosure. Participants’ preferences did not vary based on concordance or discordance between author type and disclosure. Further, the interaction between seriousness of topic and disclosure was not statistically significant (pairwise effect in eTable 3; interaction in eTable 4 in Supplement 1). There were no significant interactions between demographic factors and scenarios (eTable 4 in Supplement 1).

### AI Disclosure Verbiage Preferences

Of 1455 participants, 893 (61.4%) responded to the follow-up survey. The AI disclosure statement that received the highest proportion of most preferred (33%) was, “This message was written by Dr. T. with the support of automated tools” (eFigure 2 in Supplement 1).

## Discussion

This survey study found that participants had a slight preference for clinician messages written by AI over those written by a human, and yet participants expressed higher satisfaction with messages they were told were written by their clinician over those they were told were written by AI. While statistically significant, the difference in overall patient satisfaction was small, with more than 75% of patients expressing satisfaction regardless of the author or disclosure.

Participants preferred AI-drafted messages, which tended to be longer, included more details, and likely seemed more empathetic than human-drafted messages (eTable 1 in Supplement 1).^7^ However, regardless of the actual author, participants were overall more satisfied with messages when the disclosure informed them that their clinician had written the response or when there was no disclosure compared with a disclosure indicating AI wrote the message. This contradiction is particularly important in the context of research showing that increased access to clinicians via electronic communication improves patient satisfaction, while evidence linking the in-basket to burnout is prompting development and use of automated tools for clinicians to reduce time spent in electronic communication.^1,15^ The lack of difference in preferences between human vs no disclosure may indicate that surveyed participants assume a human author unless explicitly told otherwise.

Our results suggest patients do not experience automation bias, which refers to the tendency to prefer or trust recommendations from automated decision-making systems over those not generated by automation, even if the latter is most accurate.^16,17^ In fact, our findings may demonstrate a reverse bias in medicine, where patients prefer messages they believe come from their clinicians rather than from a model.

Interestingly, there was no interaction between author and disclosure. Based on the main effect results, we would assume that patients would have even stronger positive preferences for messages written by AI with a human disclosure and even more negative preferences for messages written by a human with an AI disclosure. However, the interaction effect was not statistically significant. Moreover, seriousness of the topic did not interact with the disclosure to change patient preferences. While we hypothesized that patients would be more accepting of messages they are told come from AI via disclosure in less serious topics, such as a medication refill, there was no interaction. We thus theorize that in this early phase of AI use in health care, patients’ comfort with AI is not context based, and they have not yet developed nuanced preferences regarding which situations represent an acceptable use of AI. These preferences may develop after users have more experiences with AI in different clinical and nonclinical situations. It is also possible that an interaction may exist but our sample was too small to detect it.

We, as clinicians and health care leaders, intend to keep using AI to address burnout and support clinicians. However, governance and oversight of ethical implementation of AI is still quickly evolving in the policy landscape.^9^ National guidelines, published by the White House as part of an AI Bill of Rights, include a section on Notice and Explanation that states, “[The patient] should know that an automated system is being used…[The patient] should know how and why an outcome impacting [them] was determined by an automated system, including when the automated system is not the sole input determining the outcome,” suggesting that any AI involvement should be disclosed.^8^ However, bioethicist Blumenthal-Barby adds further nuance to the disclosure question, arguing that mere disclosure will mean little to patients, whereas an explanation of technical details, risks, and benefits may overwhelm patients who inherently trust their clinicians to make the right decisions.^10^ Laacke et al,^18^ Nannini et al,^19^ and Jedličková,^20^ however, have argued that ethical requirements for full disclosure, and even explainability to the extent possible, stem from duties based on principles of respect for patient autonomy, regardless of the impact of such disclosure on satisfaction.

Together these findings raise several ethical and operational questions for health systems implementing AI as a tool for handling the in-basket. The operational options are (1) not to disclose the use of AI in patient communication because patients tended to be less satisfied when they were told AI was involved, or (2) to disclose, which aligns with bioethical norms and follows the White House’s AI Bill of Rights.^8^ A third option, which is ethically reasonable but practically challenging, would be to vary disclosure based on how each individual elects to receive or not receive information regarding AI.

From an ethics perspective, there is arguably more to “doing the right thing” than simply optimizing satisfaction. Patients have a right to know information relevant to their care, and the source of the information they are receiving is indeed relevant. Moreover, the power imbalance that already exists between patients and clinicians should not be exacerbated by hiding relevant aspects related to the delivery of care. If anything, AI tools should be implemented in ways that empower patients every step of the way.

Notably, it is possible that these findings reflect a time of transition into a new era of clinical norms. As AI tools become more prevalent in health care, it may be reasonable to expect that patients will become accustomed to receiving AI-generated responses and that the response author will have a smaller influence on satisfaction. This hypothesis calls for further studies that follow trends as the implementation of AI progresses.

Our findings indicate that disclosing the use of AI, in addition to aligning with ethical principles, did not lead to a large reduction in patient satisfaction. Therefore, potential patient dissatisfaction should not be viewed as a barrier to disclosure. Moreover, we found that the satisfaction, perceived usefulness, and feeling of being cared for remained high despite disclosure of AI. In our follow-up survey, we addressed the question of how best to disclose the use of AI. Participants preferred the shortest disclosure statement, which stated, “This message was written by Dr T. with the support of automated tools,” a takeaway that we are implementing at our health system. The preference toward brevity further supports Blumenthal-Barby’s hypothesis that overly complicated disclosures may overwhelm patients.^10^

Future directions include studying the reasons behind patients’ satisfaction, such as testing the inference that without disclosure, they assume a human wrote the response. Furthermore, after broader AI uptake, future studies should assess how each patient’s preferences toward AI vary between both clinical topics and nonclinical use cases to understand whether comfort with AI is situational or whether preferences are determined at the patient level.

Further, future work should explore not just whether we disclose, but how. We hope to study how the provision of additional information for patients regarding the risks and benefits of AI impacts their preferences, particularly when considering the diverse needs of different patient populations. By empowering patients with knowledge about AI in health care, we may be able to foster a more informed and collaborative approach to AI-assisted patient-physician communication.^21,22,23^

### Limitations

The study was limited by its survey-based design using a series of hypothetical topics as opposed to true patient-clinician portal messages specific to each participant covering a wider range of patient interactions. Further, the study did not assess participants’ assumptions nor reasons why certain responses were preferred. Additionally, the participant population is limited to a single health system, and respondents tended to be older, identify as non-Hispanic White, have higher educational levels, and be retired more often than our overall participant population, which may limit generalizability. Notably, this population is different than the population most familiar with generative AI, which includes more male and younger individuals.^24^ Finally, we did not explicitly ask participants about their preferences related to disclosure and instead inferred their preferences based on reported satisfaction.

## Conclusions

In this survey study, participants preferred AI-drafted messages but had slightly greater satisfaction when they were not explicitly told that AI had authored them. Participants preferred the disclosure that acknowledged AI assistance in composing the message. This, despite our finding that participants preferred AI-drafted messages, we believe health systems should disclose AI using simple terms to empower patients by reducing the information imbalance with their clinicians.

## Notes

Supplement 1. **eTable 1.** Survey Design **eTable 2.** Mean Change in Preference Scores Based Upon Variables **eTable 3.** Pairwise Effect of Scenario Seriousness **eTable 4.** Interaction Between Demographics and Domains **eTable 5.** Outcome Measures on Survey **eFigure 1.** Survey Design Example **eFigure 2.** AI Disclosure Language Preferences

Supplement 2.
Data Sharing Statement

## References

[1] Fogg JF, Sinsky CA. In-basket reduction: a multiyear pragmatic approach to lessen the work burden of primary care physicians. NEJM Catalyst. 2023;4(5):CAT.22.0438. doi:10.1056/CAT.22.0438

[2] Dyrbye LN, Gordon J, O’Horo J, . Relationships between EHR-based audit log data and physician burnout and clinical practice process measures. Mayo Clin Proc. 2023;98(3):398-409. doi:10.1016/j.mayocp.2022.10.027 36868747

[3] Senft N, Butler E, Everson J. Growing disparities in patient-provider messaging: trend analysis before and after supportive policy. J Med Internet Res. 2019;21(10):e14976. doi:10.2196/14976 31593539 PMC6803888

[4] Epic and Microsoft bring GPT-4 to EHRs. Epic. May 5, 2023. Accessed April 12, 2024. https://www.epic.com/epic/post/epic-and-microsoft-bring-gpt-4-to-ehrs/

[5] Garcia P, Ma SP, Shah S, . Artificial intelligence–generated draft replies to patient inbox messages. JAMA Netw Open. 2024;7(3):e243201. doi:10.1001/jamanetworkopen.2024.3201 38506805 PMC10955355

[6] Liu S, McCoy AB, Wright AP, . Leveraging large language models for generating responses to patient messages—a subjective analysis. J Am Med Inform Assoc. 2024;31(6):1367-1379. doi:10.1093/jamia/ocae052 38497958 PMC11105129

[7] Ayers JW, Poliak A, Dredze M, . Comparing physician and artificial intelligence chatbot responses to patient questions posted to a public social media forum. JAMA Intern Med. 2023;183(6):589-596. doi:10.1001/jamainternmed.2023.1838 37115527 PMC10148230

[8] Office of Science and Technology Policy. Blueprint for an AI Bill of Rights: making automated systems work for the American people. The White House, Office of Science and Technology Policy. 2022. Accessed January 20, 2025. https://bidenwhitehouse.archives.gov/ostp/ai-bill-of-rights

[9] Augesnstein J, Seigel R, Fox A, Shashoua M. Manatt Health: health AI policy tracker. Manatt. July 31, 2024. Accessed August 5, 2024. https://www.manatt.com/insights/newsletters/health-highlights/manatt-health-health-ai-policy-tracker

[10] Blumenthal-Barby J. An AI bill of rights: implications for health care AI and machine learning—a bioethics lens. Am J Bioeth. 2023;23(1):4-6. doi:10.1080/15265161.2022.2135875 36269302

[11] DHL survey on generative AI for MyChart messaging. ClinicalTrials.gov identifier: NCT06108037. Updated December 13, 2023. Accessed January 20, 2025. https://clinicaltrials.gov/study/NCT06108037

[12] Rosala M. Rating scales in UX research: Likert or semantic differential? June 7, 2020. Accessed April 21, 2024. https://www.nngroup.com/articles/rating-scales/

[13] Vagias WM. Likert-Type Scale Response Anchors. Clemson University; 2006.

[14] Benjamini Y, Hochberg Y. Controlling the false discovery rate: a practical and powerful approach to multiple testing. J R Stat Soc B. 2018;57(1):289-300. doi:10.1111/j.2517-6161.1995.tb02031.x

[15] Yang R, Zeng K, Jiang Y. Prevalence, factors, and association of electronic communication use with patient-perceived quality of care from the 2019 Health Information National Trends Survey 5-Cycle 3: exploratory study. J Med Internet Res. 2022;24(2):e27167. doi:10.2196/27167 35119369 PMC8857700

[16] Goddard K, Roudsari A, Wyatt JC. Automation bias: a systematic review of frequency, effect mediators, and mitigators. J Am Med Inform Assoc. 2012;19(1):121-127. doi:10.1136/amiajnl-2011-000089 21685142 PMC3240751

[17] Abbasi J, Hswen Y. Blind spots, shortcuts, and automation bias—researchers are aiming to improve AI clinical models. JAMA. 2024;331(11):903-906. doi:10.1001/jama.2023.28262 38416482

[18] Laacke S, Mueller R, Schomerus G, Salloch S. Health-related digital autonomy: a response to the commentaries. Am J Bioeth. 2021;21(10):W1-W5. doi:10.1080/15265161.2021.1965257 34554071

[19] Nannini L, Marchiori Manerba M, Beretta I. Mapping the landscape of ethical considerations in explainable AI research. Ethics Inf Technol. 2024;26(3):44. doi:10.1007/s10676-024-09773-7

[20] Jedličková A. Ethical approaches in designing autonomous and intelligent systems: a comprehensive survey towards responsible development. AI Soc. Published online August 6, 2024. doi:10.1007/s00146-024-02040-9

[21] Barkal JL, Stockert JW, Ehrenfeld JM, Cohen LK. AI and the evolution of the patient–physician relationship. In: Byren MF, Parsa N, Greenhill AT, Chahal D, Ahmad O, Bagci U, eds. AI in Clinical Medicine: A Practical Guide for Health Care Professionals. Wiley; 2023:478-487.

[22] Timmermans S. The engaged patient: the relevance of patient-physician communication for twenty-first-century health. J Health Soc Behav. 2020;61(3):259-273. doi:10.1177/0022146520943514 32723112

[23] Sass R. Equity, autonomy, and the ethical risks and opportunities of generalist medical AI. AI Ethics. Published online December 5, 2023. doi:10.1007/s43681-023-00380-8

[24] Vogels EA. A majority of Americans have heard of ChatGPT, but few have tried it themselves. Pew Research Center. May 24, 2023. Accessed September 3, 2024. https://www.pewresearch.org/short-reads/2023/05/24/a-majority-of-americans-have-heard-of-chatgpt-but-few-have-tried-it-themselves/

