# Supplementary material for: Ethics in Patient Preferences for Artificial Intelligence–Drafted Responses to Electronic Messages
Source: JAMA Netw Open. 2025 Mar 11;8(3):e250449. doi: 10.1001/jamanetworkopen.2025.0449 (PMC11897835; doi:10.1001/jamanetworkopen.2025.0449)
Supplement: Supplement 1. — eTable 1. Survey Design eTable 2. Mean Change in Preference Scores Based Upon Variables eTable 3. Pairwise Effect of Scenario Seriousness eTable 4. Interaction Between Demographics and Domains eTable 5. Outcome Measures on Survey eFigure 1. Survey Design Example eFigure 2. AI Disclosure Language Preferences [file jamanetwopen-e250449-s001.pdf]

Supplementary Online Content

Cavalier JS, Goldstein BA, Ravitsky V, et al. Ethics in patient preferences for artificial intelligence–drafted responses to electronic messages. *JAMA Netw Open.* 2025;8(3):e250449. doi:10.1001/jamanetworkopen.2025.0449

eTable 1. Survey Design
eTable 2. Mean Change in Preference Scores Based Upon Variables
eTable 3. Pairwise Effect of Scenario Seriousness
eTable 4. Interaction Between Demographics and Domains
eTable 5. Outcome Measures on Survey
eFigure 1. Survey Design Example
eFigure 2. AI Disclosure Language Preferences

This supplementary material has been provided by the authors to give readers additional information about their work.

© 2025 Cavalier JS et al. *JAMA Network Open*.

eTable 1. Survey Design

Vignette			Response Author		Disclosure
Topic	Seriousness	Patient Question	Human Response	AI Response	Options
A	Least	My pharmacy said they never received the prescription for my metformin. Could you send it again?	Sure, will send it over now.<br>Sincerely,<br>Dr. T	Of course, I apologize for any inconvenience you've experienced. I'll resend your prescription for metformin to your pharmacy right away. Please double-check with your pharmacy to ensure they receive it this time. If you encounter any further issues or have additional questions, feel free to reach out, and we'll do our best to assist you. Your health and well-being are important to us, and we appreciate your diligence in managing your medication.<br>Sincerely,<br>Dr. T	Human: "This message was created entirely by Dr. T without the use of text generation software."<br><br>or<br><br>AI: "This message was created with the help of text generation software and checked for accuracy by Dr. T before being sent."
B	Moderate	I've been using the new antibiotic for my bronchitis for a few days and I'm having a lot of nausea. I'm not sure if that is normal or not. Any advice would be	Hi. I'm sorry you are feeling nauseous. Have you had side effects from antibiotics before? If so, which ones? Are you taking them on an empty stomach? If so, taking them with food can help. Try that and see if it helps. Let me	Hello,<br>I'm sorry to hear that you're experiencing nausea while taking the new antibiotic for your bronchitis. Nausea can be a potential side effect of antibiotics, and it's essential to address it. Have you had any previous experiences with antibiotic	

© 2025 Cavalier JS et al. *JAMA Network Open*.

		greatly appreciated.	know if that doesn’t help.<br>Sincerely,<br>Dr. T	side effects? Knowing which antibiotics may have caused issues in the past can be helpful.<br>Regarding your current antibiotic, if you've been taking it on an empty stomach, this might be contributing to the nausea. I recommend trying to take the antibiotic with a meal or a light snack. Often, this simple change can alleviate the discomfort.<br>Please give the food-based approach a try and monitor if it helps with the nausea. If the symptoms persist or worsen despite this adjustment, or if you have any other concerns, please don't hesitate to contact us.<br>Your well-being is our priority, and we're here to provide support and guidance during your bronchitis treatment.<br>Sincerely,<br>Dr. T	
C	Most	I saw the results from my lung scan. It said that a 2.3 x 1.8 cm mass was found and	Hello. Pulmonary hamartomas are most commonly benign. In order to best address these questions and	Hello,<br>I understand your concern after reading the results of your lung scan. A pulmonary hamartoma is	

© 2025 Cavalier JS et al. *JAMA Network Open*.

		that the "features are highly suggestive of a pulmonary hamartoma". What does this mean? Should I be worried?	plan moving forward, I would recommend we schedule a telehealth or in person visit. I will forward this message to our schedulers to get a time set up with you.<br>Sincerely,<br>Dr. T	typically a non-cancerous growth in the lung. It's important to note that these growths are most commonly benign, which means they are usually not a cause for immediate worry. However, to provide you with a more accurate understanding of your specific situation and address any concerns you may have, I recommend that we schedule either a telehealth or in-person visit. I will forward your message to our scheduling team, and they will reach out to you soon to arrange a convenient time for your appointment. During our discussion, we can thoroughly review your scan, discuss your medical history, and develop an appropriate plan for your peace of mind and well-being. Please don't hesitate to reach out if you have any immediate questions or concerns.<br>Sincerely,<br>Dr. T	

© 2025 Cavalier JS et al. *JAMA Network Open*.

eTable 2. Mean Change in Preference Scores Based Upon Variables

	Mean score change (95% CI)		
Contrast	Satisfied	Useful	Cared
Response from Human vs AI	-0.299 (-0.368, -.230) ***	-0.281 (-0.341, -0.220) ***	-0.434 (-0.503, -0.365) ***
Disclosure to participants: From AI vs. No Disclosure	-0.091 (-0.174, -0.008) *	-0.082 (-0.155, -0.009) *	-0.157 (-0.239, -0.074) ***
Disclosure to participants: From human vs. No Disclosure	0.041 (-0.042, 0.124)	0.057 (-0.016, 0.130)	0.035 (-0.047, 0.117)
Disclosure to participants: From human vs. From AI	0.132 (0.049, 0.216)**	0.139 (0.066, 0.213)***	0.192 (0.109, 0.274) ***

* p<0.05, **p<0.01, ***<0.001

© 2025 Cavalier JS et al. *JAMA Network Open*.

eTable 3. Pairwise Effect of Scenario Seriousness

A: Least Serious, B: Moderate, C: Most Serious

	**Mean score change (95% CI)**		
**Contrast**	**Satisfied**	**Useful**	**Cared**
Scenario B vs Scenario A	0.100 (0.017, 0.183) *	0.008 (-0.065, 0.081)	0.009 (-0.073, 0.091)
Scenario C vs Scenario A	-0.044 (-0.127, 0.039)	-0.006 (-0.079, 0.067)	-0.107 (-0.190, -0.025) *
Scenario C vs Scenario B **	-0.144 (-0.228, -0.061)***	-0.014 (-0.087, 0.060)	-0.116 (-0.199, -0.034)

* p<0.05, **p<0.01, ***<0.001

© 2025 Cavalier JS et al. *JAMA Network Open*.

eTable 4. Interaction Between Demographics and Domains

P-values are adjusted using Benjamini and Hochberg correction for multiple testing.

"I am satisfied with this interaction."

	Domain	p-value for main effect for demographics	p-value for main effect: domain	Interaction: Multiple testing Adjusted p-value
Age	Scenario	0.751	0.002	0.330
	Response		< 0.001	0.833
	Disclosure		0.006	0.903
Gender	Scenario	0.544	0.002	0.727
	Response		< 0.001	0.727
	Disclosure		0.006	0.727
Highest level of education	Scenario	0.560	0.001	0.727
	Response		< 0.001	0.727
	Disclosure		0.052	0.918
Employment Status	Scenario	0.287	0.002	0.833
	Response		< 0.001	0.833
	Disclosure		0.007	0.903
Race/Ethnicity	Scenario	0.166	0.003	0.903
	Response		< 0.001	0.833
	Disclosure		0.006	0.833

"The information is useful."

	Domain	p-value for main effect for demographics	p-value for main effect: domain	Interaction: Multiple testing Adjusted p-value
Age	Scenario	0.318	0.936	0.300
	Response		< 0.001	0.925
	Disclosure		0.001	0.400
Gender	Scenario	0.612	0.935	0.525
	Response		< 0.001	0.400
	Disclosure		0.001	0.611
Highest level of education	Scenario	0.986	0.912	0.925
	Response		< 0.001	0.925
	Disclosure		0.007	0.742
Employment Status	Scenario	0.089	0.918	0.776
	Response		< 0.001	0.610

© 2025 Cavalier JS et al. *JAMA Network Open*.

	Disclosure		0.001	0.570
Race/Ethnicity	Scenario	0.284	0.928	0.925
	Response		< 0.001	0.611
	Disclosure		0.001	0.925

"I feel cared for during this interaction."

	Domain	p-value for main effect for demographics	p-value for main effect: domain	Interaction: Multiple testing Adjusted p-value
Age	Scenario	0.430	0.009	0.045
	Response		< 0.001	0.620
	Disclosure		< 0.001	0.458
Gender	Scenario	0.141	0.009	0.250
	Response		< 0.001	0.250
	Disclosure		< 0.001	0.458
Highest level of education	Scenario	0.758	0.019	0.458
	Response		< 0.001	0.854
	Disclosure		0.001	0.854
Employment Status	Scenario	0.017	0.008	0.458
	Response		< 0.001	0.397
	Disclosure		< 0.001	0.805
Race/Ethnicity	Scenario	0.270	0.010	0.642
	Response		< 0.001	0.854
	Disclosure		< 0.001	0.850

eTable 5. Outcome Measures on Survey

Prompt	Please indicate your level of agreement with the following questions, using the scale below.				
Questions	1. I am satisfied with this interaction.				
	2. The information is useful.				
	3. I feel cared for during this interaction.				
Response Options	1	2	3	4	5
	Strongly Disagree	Disagree	Neither agree nor disagree	Agree	Strongly Agree

© 2025 Cavalier JS et al. *JAMA Network Open*.

eFigure 1. Survey Design Example

Images below show how the surveys appeared to participants. This example is from the third of three surveys with this participant receiving the moderate seriousness topic with the human response and the artificial intelligence disclosure. Example showcases a discordant example scenario in which the actual author did not match the disclosure.

Page 1 Example

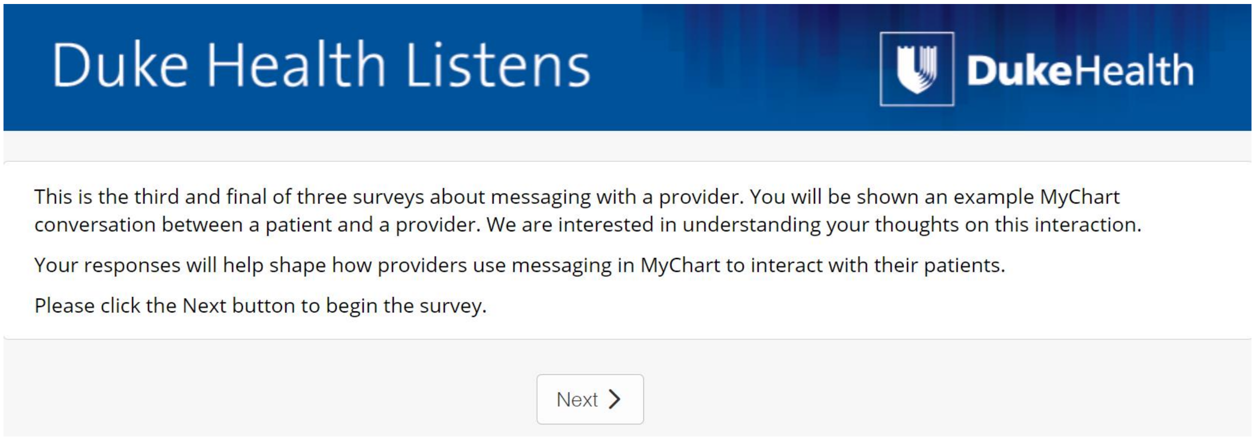

Page 2 Example

Duke Health Listens

DukeHealth

You send the following message to your physician, Dr. T:

"I've been using the new antibiotic for my bronchitis for a few days and I'm having a lot of nausea. I'm not sure if that is normal or not. Any advice would be greatly appreciated."

You receive the following reply:

"Hi, I'm sorry you are feeling nauseous. Have you had side effects from antibiotics before? If so, which ones? Are you taking them on an empty stomach? If so, taking them with food can help. Try that and see if it helps. Let me know if that doesn't help."

Sincerely,

Dr. T"

This message was created with the help of text generation software and checked for accuracy by Dr. T before being sent.

Please indicate your level of agreement with the following questions.

* I am satisfied with this interaction.

Strongly Disagree	Disagree	Neither agree nor disagree	Agree	Strongly Agree

* The information is useful.

Strongly Disagree	Disagree	Neither agree nor disagree	Agree	Strongly Agree

* I feel cared for during this interaction.

Strongly Disagree	Disagree	Neither agree nor disagree	Agree	Strongly Agree

Next >

© 2025 Cavalier JS et al. *JAMA Network Open*.

Page 3 Example

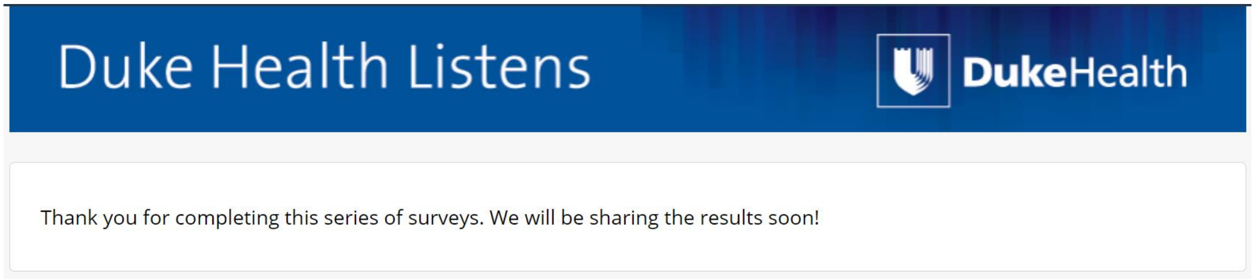

eFigure 2. AI Disclosure Language Preferences

In a follow-up survey, participants were asked, “For the following disclosures, please rank which of the explanations you prefer most (#1) to least (#5) in terms of how comfortable you feel with Dr. T’s use of automated tools.” Figure shows ranked preferences from 893 respondents.

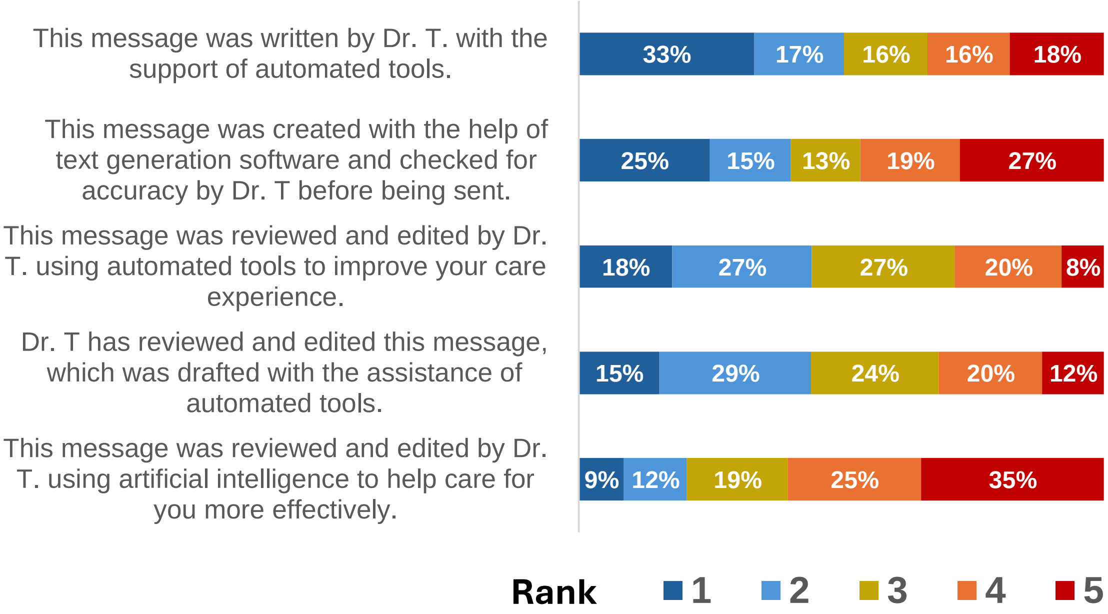

© 2025 Cavalier JS et al. *JAMA Network Open*.
