# Supplementary material for: Ethics in Patient Preferences for Artificial Intelligence–Drafted Responses to Electronic Messages
Source: JAMA Netw Open. 2025 Mar 11;8(3):e250449. doi: 10.1001/jamanetworkopen.2025.0449 (PMC11897835; doi:10.1001/jamanetworkopen.2025.0449)
Supplement: Supplement 2. — Data Sharing Statement [file jamanetwopen-e250449-s002.pdf]

Data Sharing Statement

Cavalier. Ethics in Patient Preferences for Artificial Intelligence–Drafted Responses to Electronic Messages. *JAMA Netw Open*. Published March 11, 2025.
doi:10.1001/jamanetworkopen.2025.0449

Data
Data available: No

Additional Information
Explanation for why data not available: Patients voluntarily joined the patient advisory panel used for this study and consented to receive surveys such as ours. Since they did not specifically consent to data sharing, we will not be able to share our source data.
